# Mysteries in embryonic development: How can errors arise so frequently at the beginning of mammalian life?

**DOI:** 10.1371/journal.pbio.3000173

**Published:** 2019-03-06

**Authors:** Isabell Schneider, Jan Ellenberg

**Affiliations:** 1 Cell Biology and Biophysics Unit, European Molecular Biology Laboratory, Heidelberg, Germany; 2 Candidate for joint PhD degree between EMBL and Heidelberg University, Faculty of Biosciences, Heidelberg, Germany

## Abstract

Chromosome segregation errors occur frequently during female meiosis but also in the first mitoses of mammalian preimplantation development. Such errors can lead to aneuploidy, spontaneous abortions, and birth defects. Some of the mechanisms underlying these errors in meiosis have been deciphered but which mechanisms could cause chromosome missegregation in the first embryonic cleavage divisions is mostly a “mystery”. In this article, we describe the starting conditions and challenges of these preimplantation divisions, which might impair faithful chromosome segregation. We also highlight the pending research to provide detailed insight into the mechanisms and regulation of preimplantation mitoses.

## Abstract 2

Starting a new life is a challenging business. This Essay explores the changes at the oocyte-to-embryo transition to highlight the circumstances under which the very first and decisive — but ‘mysteriously’ error-prone — mitotic divisions occur.

## Background

When chromosomes segregate erroneously in oocyte meiosis or in mitosis during preimplantation development, the effects can be detrimental for the embryo and the course of pregnancy because these errors can lead to aneuploidies, spontaneous abortions, and birth defects. Studies on mammalian fertility indicated very soon that fundamental problems must occur during preimplantation development. A study in the 1950s found that only approximately 58% of naturally conceived embryos were able to implant in the uterus at blastocyst stage [1]. Subsequently, many studies examining oocytes and early embryos from several mammalian species, including human oocytes and embryos from patients undergoing assisted reproductive treatment, have provided clear evidence that the division fidelity of female meiosis and embryonic mitoses is substantially lower than in cells of somatic tissues [2–4].

The meiotic divisions of the oocyte are very different from mitotic divisions of somatic cells: the diploid genome has to be reduced to allow for complementation by the haploid genome of the sperm delivered at fertilization. Chromosomes in the oocyte are therefore segregated twice without intermediate replication. In addition, the large oocyte cleaves asymmetrically. To retain most of the stored cytoplasmic material in the mature egg to nurture the embryo, the oocyte extrudes half of the chromosomes into a small nonfertilizable and unviable polar body at each meiotic division.

In meiosis I, homologous chromosomes are split. To avoid that sister chromatids separate prematurely, in most eukaryotic species, kinetochores either fuse or juxtapose side by side [5]. Additionally, the homologues have to be paired and physically linked by crossovers of their DNA for faithful segregation. And finally, stable cohesion at centromeres of the sister chromatids ensures that entire homologues get pulled to opposite spindle poles by kinetochore-attached microtubules [5,6]. On the contrary, in meiosis II and the later mitoses, the two replicated sister chromatids of single chromosomes have individualized kinetochores and are only joined by cohesin rings until anaphase. Therefore, the sister chromatids can become individually attached and segregated to opposite spindle poles.

The very different nature of meiosis I is thought to be the source of most of the errors that occur during maturation of the oocyte to a fertilizable egg. Evidence comes from studies of eggs from mice and from women undergoing assisted reproductive procedures: they show that failure to link the homologous chromosomes and premature separation of sister kinetochores mainly drive mammalian oocyte aneuploidy, because here, sister chromatids segregate precociously, and these events seem to strongly increase with maternal age [7–10].

Segregation errors even occur after the egg has been fertilized. Studies of mammalian preimplantation embryos have shown that blastomeres of different genomic content are abundant [3,11,12], suggesting that chromosomes also frequently missegregate during the mitotic cleavage divisions after fertilization. Such mosaic chromosome abnormalities can vary from a single cell to all cells in the embryo, and individual cells of the same embryo can exhibit different chromosomal compositions. This is ruling out a sole carryover of aneuploidy resulting from meiotic errors [11]. Therefore, even if oocytes mature normally and become fertilized, the first embryonic mitoses are also error prone, which can affect normal development or lead to abortion. A clinical study has shown that some human mosaic blastocysts can implant and the embryo develop to term without genetic disorder. The authors suggested that the survival depends on the type and extent of mosaicism [13]. A recent study using a mouse model for embryonic mosaicism supports this hypothesis, indicating that a minimal number of euploid blastomeres is necessary for normal embryonic development [14]. Increased apoptosis was observed for the abnormal cells within mosaic embryos, and cell competition could be another potential mechanism for the embryo to cope with aneuploid cells [14,15]. However, because of its mosaic nature and cell-to-cell variability, embryonic aneuploidy poses a bigger challenge for in vitro fertilization procedures and assessment of embryonic quality, even if genetic preimplantation diagnostics are used. Genome sequencing of a single cell from an eight-cell blastomere or of a blastocyst biopsy prior to transferring the embryo into the uterus might show a normal genome but may not be representative for the variable genomic content in different cells of the embryo [16,17].

But how and why do these errors occur in the first mitotic divisions despite these divisions being decisive for the healthy start of a new life? Why does the embryo proceed with the cleavage divisions before correcting chromosome segregation errors? To solve this mystery, it is crucial to understand early embryonic mitoses in all of their complexity, especially how the structural and regulatory molecules differ from faithful somatic cell mitosis. Due to the limited availability and technical difficulty of studying mammalian embryos, molecular mechanistic studies are still very sparse. We only start to unravel the nature of the early embryonic mitotic machinery and its regulation. But that should give us clues as to why the beginning of mammalian life is so risky, and how we can improve procedures to reliably recognize and mitigate these risks in fertility treatments.

## Early embryonic mitoses in mammals—Turning a large slowly dividing egg into small rapidly dividing somatic cells

Mitosis of somatic cells during postimplantation development and in adult tissue homeostasis and regeneration is typically fast, produces two cells of similar size, and has faithful chromosome segregation, with error rates of below 5% in adult murine and human tissue [2]. These short mitoses rely on fast and efficient spindle assembly with two centrosomes, which strongly nucleate microtubules and predetermine spindle bipolarity. This leads to relatively few chromosome biorientation errors. In addition, the spindle assembly checkpoint (SAC) ensures that chromosomes are faithfully segregated: it delays anaphase onset until all kinetochores are properly attached, typically achieved in only a few minutes [18].

**Fig 1:**
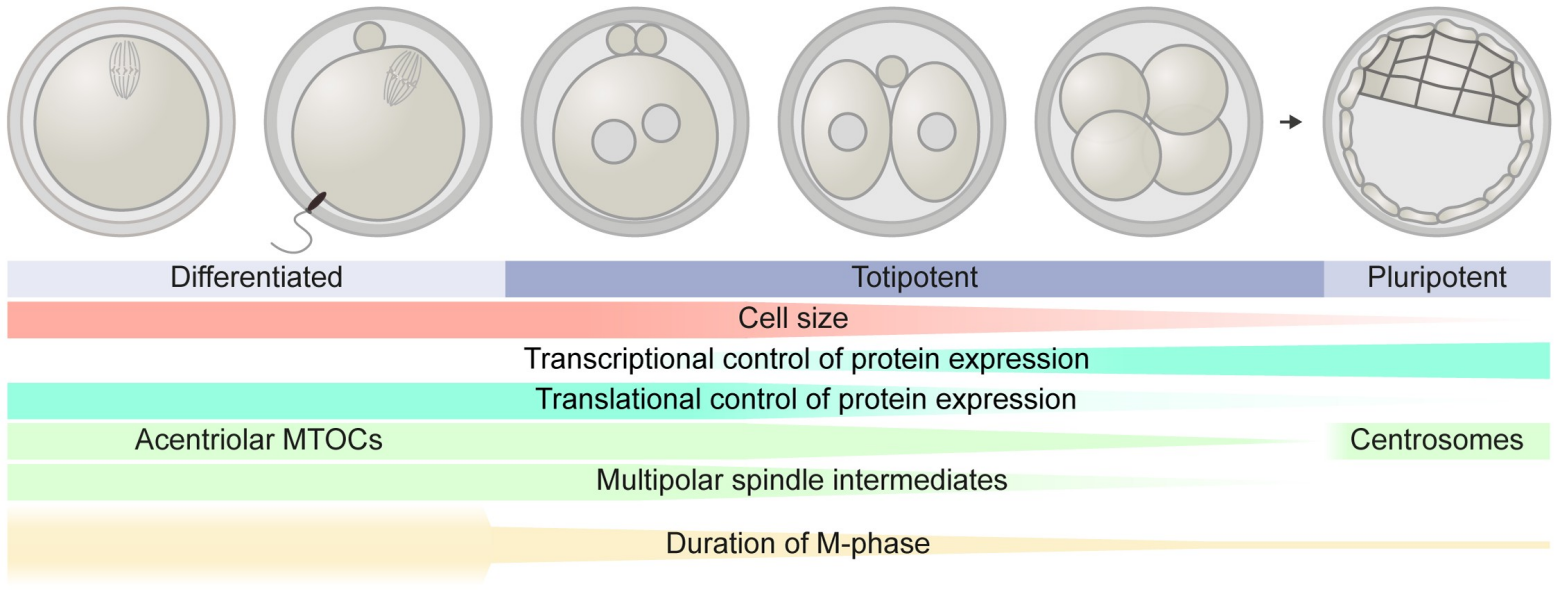
Gradual changes during mouse preimplantation development could affect chromosome segregation fidelity. After meiosis I, the fully differentiated oocyte is fertilized by the sperm and resumes meiosis II. The two divisions are very asymmetric to maintain a large cytoplasmic volume for storage. During both meiotic divisions, transcription is inactive and protein expression is translationally and post-translationally regulated. Because centrosomes are degraded during early oogenesis [26], multiple acentriolar MTOCs functionally replace them in meiotic spindle assembly [19,27]. Thereby, multipolar spindle intermediates preferentially form during prometaphase. Also the duration of the division phase in the cell cycle (M-phase) is very long: meiosis I can take up to 10 hours and meiosis II can be especially delayed due to a metaphase arrest until fertilization [28]. Therefore, the totipotent zygote undergoes the first mitosis under unusual conditions, and the described and depicted parameters only gradually change during the early divisions: in every cleavage, the cell size is halved, whereas the genomic content should remain constant [25]. Through stored and activated mRNA, the zygote is still entirely under maternal translational control [23]. Only from the two-cell stage onwards—when transcription of the embryonic genome becomes activated—maternal control gets replaced by embryonic control [29]. Fewer and fewer MTOCs organize the mitotic spindle until centrosomes form de novo at the blastocyst stage. Until then, spindle assembly passes through less marked multipolar organization [30,31]. Also, M-phase duration changes: after very lengthy and even halted meiosis, the zygotic mitosis is still prolonged (approximately 90 to 120 minutes) compared to somatic mitosis [32,33]. Thereafter, the duration decreases until it reaches the timing of a normal somatic cell mitosis. These gradual changes during preimplantation development might contribute to decreased chromosome segregation fidelity during cleavage divisions. MTOC, microtubule organizing center.

At the beginning of mammalian life, the conditions for the first mitotic cell divisions are fundamentally different (Fig 1). Except for the paternal genome, almost all molecular material for the zygotic division is contributed maternally, using the proteins and mRNAs stored in the egg. Until fertilization, these molecules mediated the first and part of the second meiotic division of the oocyte, both of which are slow, error prone, and asymmetric. Here, spindle assembly proceeds without centrosomes, which are eliminated during early oogenesis in mammals. Instead, the meiotic spindle assembles by lengthy multipolar self-organization processes [19,20], which entail many erroneous biorientation attempts [20,21]. However, the meiotic division proceeds slowly, providing several hours for error correction and spindle migration for symmetry breaking.

At fertilization, a dramatic change occurs. The egg becomes activated when the intracellular calcium increases, which affects many cellular pathways [22]. Calcium induces a rapid change in the zona pellucida around the zygote to prevent fertilization by more than one sperm, triggers remodeling of the sperm chromatin, and induces resumption of female meiosis II [23]. Together, sperm remodeling and segregation of the maternal chromosomes facilitate the formation of two nuclei in the zygote, one for the paternal and one for the maternal genome. In these so-called pronuclei, the two haploid genomes are then replicated separately and reprogrammed to turn the highly specialized egg and sperm cells into a totipotent zygote.

The first embryonic division therefore begins in a very unusual situation (Fig 1). The zygote inherits its size and molecular material from the meiotic oocyte, whereas the parental genomes are transcriptionally silent. In addition, the zygote is in a state of transition, in which the cellular structures and regulatory pathways of the specialized meiotic divisions have to be repurposed for mitotic divisions. To achieve this, only post-transcriptional mechanisms can be used; namely, activation and translation of maternal mRNAs, as well as post-translational modifications of cytoskeletal proteins, which allow the skeleton to remodel [16]. The embryonic genome itself is only activated later in development, in mice, for example, at the two-cell stage [24]. Moreover, the zygote contains the two separate nuclei, one for each parental genome, which have to be dealt with by the mitotic apparatus. And even after the zygotic division, each of the following five divisions of preimplantation development has its own unusual circumstances. Every cleavage cuts the cell size in half because there is no interphase growth [25]. Therefore, the cytoplasmic content is reduced until genome activation and subsequent gene expression catch up. The genome, on the other hand, duplicates in each cell cycle, and thus genome size per cell remains constant. In the very first divisions, this most likely leads to the challenge to scaling fewer components of the mitotic machinery into a shrinking cytoplasmic volume to the same amount of DNA. How mitotic mechanisms and their regulation adapt to this cellular reorganization from zygote to blastocyst and how these changes are linked to the abundance of chromosome segregation errors is currently a “mystery”. Below, we discuss two major aspects of cell division that undergo fundamental changes in the preimplantation embryo, and which are likely to play a role in causing chromosomal aberrations or complicating their correction at the beginning of mammalian life: assembly of the mitotic spindle and regulation of mitotic duration.

## From multipolar acentrosomal to bipolar centrosomal spindles

In somatic cell mitosis, assembly of the bipolar spindle in prometaphase is efficiently driven by two centrosomes, powerful microtubule organizing centers (MTOCs) with a pair of centrioles at their core. However, centrioles are actively eliminated during mammalian oogenesis at the pachytene stage, and spindles are therefore acentriolar during female meiosis [26]. Consequently, mammalian oocytes assemble their first bipolar microtubule systems through self-organization mechanisms often via multipolar intermediates [19,20]. In mouse oocytes, multiple acentriolar cytoplasmic MTOCs are generated by fragmentation during late prophase. They then assemble into two broad poles of a bipolar spindle but only after transiently organizing multipolar spindle intermediates [19,27]. Also in human oocytes, even after the lengthy chromosome driven spindle assembly without MTOCs, the bipolar spindle is often unstable and can transiently become apolar or multipolar [20]. Such a situation puts kinetochores at high risk to become wrongly attached to microtubules, and thus chromosomes fail to biorient in metaphase and often lag behind the poleward movement in anaphase [20,21].

If similar multipolar spindle assembly mechanisms operated also after fertilization, they could underlie embryonic aneuploidies. In most mammalian species, however, centrioles are delivered by the sperm, and they could restore a predetermined bipolar situation. But even here, it is not entirely clear how the sperm’s centrioles are inherited and duplicated and how they contribute to organizing the first mitotic spindle [34,35]. Especially in humans, it remains controversial whether only one or both centrioles are transmitted because the distal centriole in the sperm is degenerated and used as the basis of the flagellum [36]. Studies on human zygotes using transmission electron microscopy have clearly indicated that the proximal centriole is transmitted by the sperm, but they have failed to demonstrate that centrioles are present at both poles of the first mitotic apparatus in monospermic zygotes [37,38]. Recent work has shown that distal human centrioles can function as an MTOC in *Xenopus* egg extract [39]. It is, however, unknown whether one or both sperm centrioles are sufficiently active in the human zygote to immediately organize a bipolar spindle and thereby avoid multipolar intermediates. In mice and other rodents, the sperm basal body is degraded after fertilization, making the early embryonic divisions acentriolar until centrioles, and thus centrosomes, are formed de novo at the blastocyst stage [30]. In this situation, the first cleavages still exhibit the multipolar spindle intermediates [31], which could contribute to the high abundance of chromosome segregation errors. With each cleavage division, spindle poles become gradually better focused until centrioles are formed (Fig 1). In parallel to this gradual change from multi- to bipolar spindle assembly, the amount and composition of MTOCs gradually changes, and different motors become important for spindle assembly and bipolarization [31,40]. Although the mouse is the best-characterized mammalian species for embryonic spindle assembly to date, even here, only very little is known about the mechanisms that drive the transition from multipolar and acentriolar to centrosomal spindles with predetermined spindle bipolarity. For example, what triggers the expression of centriolar proteins and their de novo assembly into centrioles at the blastocyst stage remains elusive.

**Fig 2:**
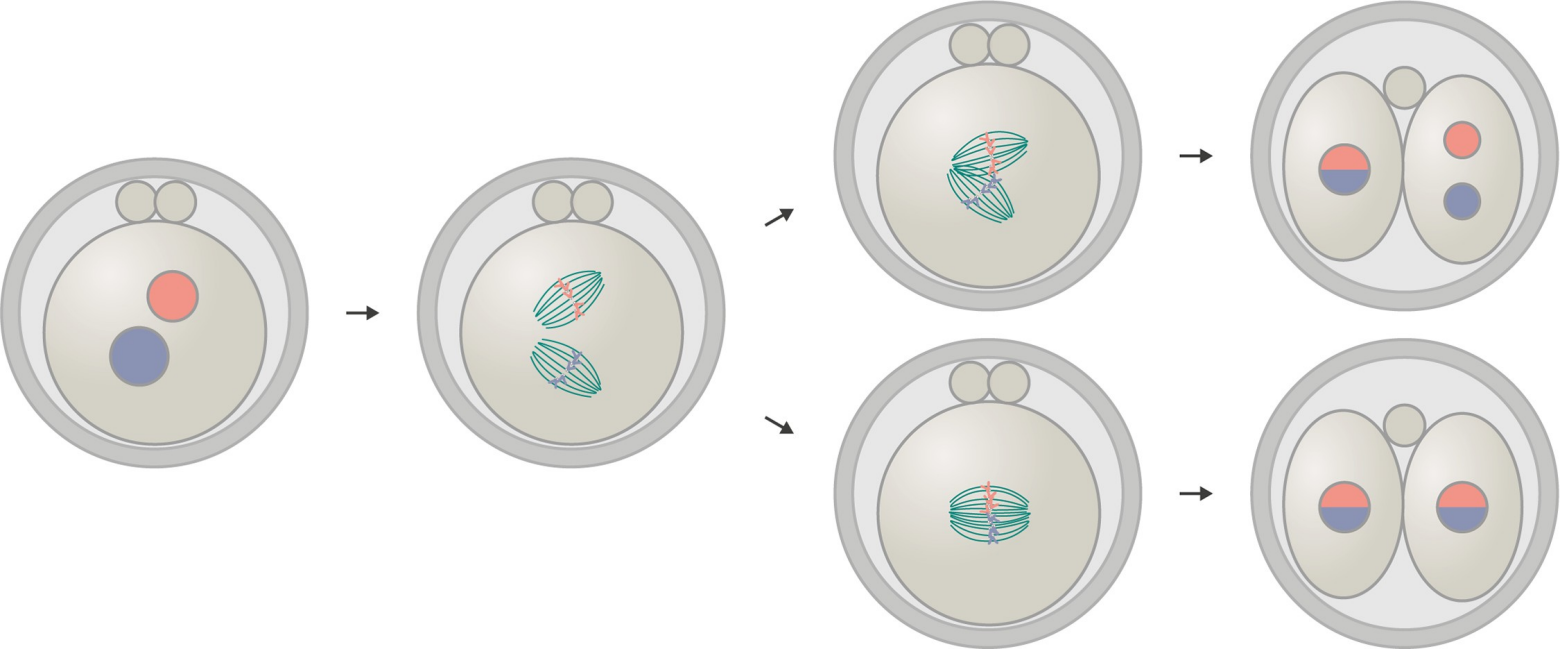
Misalignment of the two spindles in murine zygotic mitosis can result in binucleated blastomeres. In murine zygotic mitosis, two independent spindles assemble around the parental pronuclei [42]. If these two spindles fail to align in parallel prior to anaphase—e.g., in case the pronuclei are too far apart from each other at mitotic entry—two-cell embryos with blastomeres containing two nuclei can form.

Embryonic spindle assembly recently became better understood for the zygotic division in the mouse. At first, the pronuclei carrying the maternal and paternal chromosomes are brought towards the center of the zygote through F-actin–dependent movement prior to nuclear envelope breakdown [41]. Then, the mitotic apparatus is constructed in a surprising way in this cell with two nuclei (Fig 2): Not one common spindle but two spindles self-organize around the pronuclei. Each of these two spindles can bipolarize and start to congress chromosomes independently. Only later in prometaphase do they align their axes in one direction and finally function as a reasonably synchronized system that segregates the two parental chromosome sets [42]. In the mouse zygote, multipolar spindle assembly thus persists but does not build one, but two, bipolar systems. Because these two bipolar systems need to be aligned, this additional step introduces a new risk of errors. And indeed, if the two spindles fail to align, two-cell embryos can form, the blastomeres of which contain two nuclei rather than one [42] (Fig 2), which is detrimental for the next embryonic division.

## The SAC and cell cycle control at the oocyte to embryo transition

Not only spindle assembly and function, but also the control of faithful chromosome segregation and duration of the cell division phase (M-phase), are different in the oocyte and early embryo compared to somatic cells (Fig 1). In all cells, M-phase entry is governed by an increase in cyclin-dependent kinase 1 (CDK1) activity, which is mediated by synthesis and binding of cyclin B and activating the kinase through a change in its phosphorylation state [43]. Conversely, exiting M-phase requires that CDK1 activity decreases. This is mediated by degradation of cyclin B and reverse phosphoregulation of the kinase. For cyclin B to be degraded, the E3 ligase anaphase promoting complex or cyclosome (APC/C) polyubiquitinylates the protein and thereby targets it for proteasomal degradation. Upon activation of the APC/C through binding of cell division cycle protein 20 (Cdc20), the APC/C modifies key substrates, such as cyclin B and securin with ubiquitin chains, and thereby drives progression into anaphase.

In somatic cells, mitotic duration and cyclin B–dependent CDK1 activity is kept short, typically lasting only approximately 40 minutes [44,45]. Only under certain conditions can CDK1 activity be stabilized. This is primarily mediated through the SAC, which delays anaphase onset until all kinetochore pairs from sister chromatids are stably attached to spindle microtubules from opposite spindle poles [46,47]. The SAC thereby ensures that chromosomes segregate faithfully. Molecularly, the SAC achieves this by blocking APC/C activation; when kinetochores are unattached, they recruit SAC components—such as mitotic arrest deficient 2 (Mad2), budding uninhibited by benzimidazoles 3 (Bub3), and budding uninhibited by benzimidazoles-related 1 (BubR1)—that, together with Cdc20, form the mitotic checkpoint complex, which even binds a second Cdc20 molecule. Sequestering this activator of the APC/C, the SAC delays cyclin B degradation and CDK1 inactivation and thereby delays cell cycle progression into anaphase. Once all sister chromatids are correctly bioriented to microtubules from opposite spindle poles, the kinetochores stop recruiting SAC components, switching the checkpoint off [48,49].

Compared to somatic cells, M-phase in mouse oocyte meiosis I is much longer, lasting up to 10 hours. Starting with oocyte maturation, cyclin B levels increase only gradually when the protein becomes translated from previously stored and silenced mRNA [50–52]. Consequently, CDK1 activity also increases gradually. This gradual increase as well as the lengthy M-phase are thought to be essential to generate stable microtubule–kinetochore attachments in the oocyte [53]. Also, the control of faithful chromosome segregation in meiosis I differs from the control in somatic mitosis: the SAC components are present, and the checkpoint is generally active [54–58] but it appears to be less sensitive, because it can be silenced even in the presence of misaligned or misattached kinetochores, which can lead to aneuploidy [59–62]. Recent studies suggest that components of the SAC might not be sufficiently concentrated in the huge volume of the oocyte to arrest cell cycle progression in response to one or very few misattached kinetochores [63].

The SAC is also present in meiosis II [55], but its functionality remains elusive, because meiotic resumption primarily depends on the so-called cytostatic factor (CSF) [64]. CSF activity raises and stabilizes CDK1 activity after resumption of meiosis I and maintains an arrest in metaphase II until fertilization, while the SAC is silent and the kinetochores are bioriented [28]. Early mitotic inhibitor 2 (Emi2) is a key player in CSF activity and is thought to function as an inhibitor of the APC/C because it also binds to Cdc20 [65]. At fertilization, Emi2 becomes degraded, which is thought to contribute to the activation of the APC/C and consequently to degradation of cyclin B and securin, which allows progression into anaphase of meiosis II [66].

The zygote therefore inherits a cell cycle regulatory system from two meiotic divisions that either overrides the SAC or has an additional downstream mechanism to halt M-phase exit until fertilization. Zygotic cell cycle progression relies solely on stored maternal factors, as shown by CDK1 activation that is independent from the nuclei [67]. Also similar to meiosis, the first embryonic M-phase is unusually long compared to somatic mitoses, in line with the observed stabilization of CDK1 activity [32]. A recent study indicates that APC/C activation is delayed in the first M-phase compared to M-phase in the two-cell division and that this delay seems to be independent of Emi2 [33]. The increased duration of mitosis in the zygote is not caused by the dual spindle assembly process, because M-phase is not shortened in parthenogenic haploid “zygotes” [68]. Whether the prolonged zygotic M-phase is required for other events of the first embryonic cleavage, or whether it is a remnant of factors (other than Emi2) mediating the arrest in meiosis II, remains to be elucidated.

We still do not understand in detail how the cell cycle is controlled during early embryogenesis. It is clear that it changes massively from early to late preimplantation development: At the beginning, during the first lengthy cell cycles, checkpoints are most likely active. However, at blastocyst stage, the gap phases and some checkpoints of the cell cycle are skipped to allow for a massive burst in cell division [69]. But comparative studies of the cell cycle control machinery between the different cleavage divisions are still to be conducted. It is also unclear how stable microtubule kinetochore attachments and biorientation are established and controlled during the oocyte to embryo transition. From the available studies of mouse and human embryos, it seems that the SAC components are present and in principle functional during preimplantation cleavages [70,71]. Moreover, the SAC effector proteins, Bub3, Mad2, and BubR1, are essential for early embryogenesis as null mutants of these components die at very early stages of embryogenesis [72–74]. One study claims that Mad2 displaces from kinetochores already before a metaphase plate is established, indicating that the SAC might not be responsible for the prolonged M-phase in the first embryonic division [75]. But so far, we do not understand in detail how M-phase prolongation is achieved in the very first division. Systematic studies that analyze SAC activity and mitotic exit regulation and their potential impact on the fidelity of chromosome segregation in the first and subsequent embryonic mitoses are missing.

## The many risks imposed by the transition from zygote to blastocyst

A gradual transition from the “oocyte-like” zygote to the “ready-to-implant” embryo with a fully somatic mitosis might actually be the key to unlock many secrets about preimplantation cell biology and aneuploidy formation (Fig 1). When the egg is activated, post-translational remodeling of the cytoskeleton and priming of mRNAs for translation contribute to prepare the zygote for the very first mitosis. However, as described above, the zygotic division still exhibits many features of meiosis, such as risky multipolar spindle intermediates and the long duration. From the two-cell stage onwards, blastomere divisions start with one nucleus in which the transcription of the embryonic genome is then activated. Consequently, the blastomeres start to express proteins of the somatic mitotic machinery and regulation de novo. At the same time, the cleavage divisions start to speed up as cell numbers have to be increased to allow appropriate amounts of cells to differentiate into the first three cell lineages prior to implantation. Firstly, the trophectoderm, which contributes to the placenta, is essential for the blastocyst to attach to the uterine endometrium. The primitive endoderm that mainly forms the extraembryonic yolk sac has to form as well. And for the epiblast, which produces all fetal cells, a critical cell number is essential for implantation and development till birth [76]. The blastomere cell cycles after the two-cell stage therefore become relatively short compared to the time required for transcription, translation, and protein turnover. Therefore, completing the transition from the oocyte to the embryo proteome might require several cell generations. This delay in the establishment of faithfully regulated somatic mitosis in preimplantation development likely accounts for the increased frequency of chromosome missegregation.

Although some of the mechanisms contributing to aneuploidy in meiosis of mammalian oocytes have been described at the molecular level, detailed mechanistic information about the divisions and aneuploidy formation in the early embryo is almost entirely missing. Due to the constant changes in cellular state during preimplantation development, the division mechanisms might be very transient. Therefore, the molecular sources of errors might vary between the different cleavages. New technologies such as high resolution real-time observation by light-sheet microscopy [77], fast genetic perturbations by genome editing in embryos [78], and single cell sequencing now provide us with powerful tools to unravel the dynamic cellular mechanisms that explain why our lives start with such awry divisions. Once molecularly understood, comparing the situation in mammals with other vertebrates should also allow us to rationalize this puzzling fact in the light of evolution.

## Abbreviations

APC/C: anaphase promoting complex or cyclosome
Bub3: budding uninhibited by benzimidazoles 3
BubR1: budding uninhibited by benzimidazoles-related 1
Cdc20: cell division cycle protein 20
CDK1: cyclin-dependent kinase 1
CSF: cytostatic factor
Emi2: early mitotic inhibitor 2
Mad2: mitotic arrest deficient 2
MTOC: microtubule organizing center
SAC: spindle assembly checkpoint

## References

[1] Hertig AT, Rock J, Adams EC, Menkin MC. Thirty-four fertilized human ova, good, bad and indifferent, recovered from 210 women of known fertility: a study of biologic wastage in early human pregnancy. Pediatrics. 1959;23: 2002–211. 13613882

[2] Knouse KA, Wu J, Whittaker CA, Amon A. Single cell sequencing reveals low levels of aneuploidy across mammalian tissues. Proc Natl Acad Sci. National Academy of Sciences; 2014;111: 13409–13414. 10.1073/pnas.1415287111 25197050 PMC4169915

[3] Mantikou E, Wong KM, Repping S, Mastenbroek S. Molecular origin of mitotic aneuploidies in preimplantation embryos. Biochimica et Biophysica Acta—Molecular Basis of Disease. 2012 pp. 1921–1930. 10.1016/j.bbadis.2012.06.013 22771499

[4] Nagaoka SI, Hassold TJ, Hunt PA. Human aneuploidy: mechanisms and new insights into an age-old problem. Nat Rev Genet. Nature Publishing Group; 2012;13: 493–504. 10.1038/nrg3245 22705668 PMC3551553

[5] Watanabe Y. Geometry and force behind kinetochore orientation: lessons from meiosis. Nat Rev Mol Cell Biol. Nature Publishing Group; 2012;13: 370–382. 10.1038/nrm3349 22588367

[6] Nasmyth K. A meiotic mystery: How sister kinetochores avoid being pulled in opposite directions during the first division. BioEssays. John Wiley & Sons, Ltd; 2015;37: 657–665. 10.1002/bies.201500006 25874377 PMC4683677

[7] Zielinska AP, Holubcova Z, Blayney M, Elder K, Schuh M. Sister kinetochore splitting and precocious disintegration of bivalents could explain the maternal age effect. Elife. 2015;4: 1–19. 10.7554/eLife.11389 26670547 PMC4755749

[8] Angell R. Predivision in human oocytes at meiosis I: a mechanism for trisomy formation in man. Hum Genet. Springer-Verlag; 1991;86: 383–387. 10.1007/BF00201839 1999340

[9] Patel J, Tan SL, Hartshorne GM, McAinsh AD. Unique geometry of sister kinetochores in human oocytes during meiosis I may explain maternal age-associated increases in chromosomal abnormalities. Biol Open. Company of Biologists; 2015;5: 178–84. 10.1242/bio.016394 26718930 PMC4823989

[10] Sakakibara Y, Hashimoto S, Nakaoka Y, Kouznetsova A, Höög C, Kitajima TS. Bivalent separation into univalents precedes age-related meiosis I errors in oocytes. Nat Commun. Nature Publishing Group; 2015;6: 7550 10.1038/ncomms8550 26130582 PMC4507009

[11] Delhanty JDA. Mechanisms of aneuploidy induction in human oogenesis and early embryogenesis. Cytogenet Genome Res. 2005;111: 237–244. 10.1159/000086894 16192699

[12] Vázquez-Diez C, Yamagata K, Trivedi S, Haverfield J, FitzHarris G. Micronucleus formation causes perpetual unilateral chromosome inheritance in mouse embryos. Proc Natl Acad Sci. National Academy of Sciences; 2016;113: 626–631. 10.1073/pnas.1517628112 26729872 PMC4725495

[13] Greco E, Minasi MG, Fiorentino F. Healthy Babies after Intrauterine Transfer of Mosaic Aneuploid Blastocysts. N Engl J Med. Massachusetts Medical Society; 2015;373: 2089–2090. 10.1056/NEJMc1500421 26581010

[14] Bolton H, Graham SJL, Van der Aa N, Kumar P, Theunis K, Fernandez Gallardo E, et al Mouse model of chromosome mosaicism reveals lineage-specific depletion of aneuploid cells and normal developmental potential. Nat Commun. Nature Publishing Group; 2016;7: 11165 10.1038/ncomms11165 27021558 PMC4820631

[15] Bowling S, Di Gregorio A, Sancho M, Pozzi S, Aarts M, Signore M, et al P53 and mTOR signalling determine fitness selection through cell competition during early mouse embryonic development. Nat Commun. Nature Publishing Group; 2018;9: 1763 10.1038/s41467-018-04167-y 29720666 PMC5932021

[16] Gleicher N, Vidali A, Braverman J, Kushnir VA, Barad DH, Hudson C, et al Accuracy of preimplantation genetic screening (PGS) is compromised by degree of mosaicism of human embryos. Reprod Biol Endocrinol. BioMed Central; 2016;14: 54 10.1186/s12958-016-0193-6 27595768 PMC5011996

[17] Gleicher N, Metzger J, Croft G, Kushnir VA, Albertini DF, Barad DH. A single trophectoderm biopsy at blastocyst stage is mathematically unable to determine embryo ploidy accurately enough for clinical use. Reprod Biol Endocrinol. BioMed Central; 2017;15: 33 10.1186/s12958-017-0251-8 28449669 PMC5408377

[18] Pereira AJ, Maiato H. Maturation of the kinetochore-microtubule interface and the meaning of metaphase. Chromosom Res. Springer Netherlands; 2012;20: 563–577. 10.1007/s10577-012-9298-8 22801775

[19] Schuh M, Ellenberg J. Self-Organization of MTOCs Replaces Centrosome Function during Acentrosomal Spindle Assembly in Live Mouse Oocytes. Cell. 2007;130: 484–498. 10.1016/j.cell.2007.06.025 17693257

[20] Holubcová Z, Blayney M, Elder K, Schuh M. Error-prone chromosome-mediated spindle assembly favors chromosome segregation defects in human oocytes. Science. 2015;348: 1143–7. 10.1126/science.aaa9529 26045437 PMC4477045

[21] Kitajima TS, Ohsugi M, Ellenberg J. Complete kinetochore tracking reveals error-prone homologous chromosome biorientation in mammalian oocytes. Cell. 2011;146: 568–581. 10.1016/j.cell.2011.07.031 21854982

[22] Ducibella T, Huneau D, Angelichio E, Xu Z, Schultz RM, Kopf GS, et al Egg-to-Embryo Transition Is Driven by Differential Responses to Ca2+ Oscillation Number. Dev Biol. Academic Press; 2002;250: 280–291. 10.1006/DBIO.2002.0788 12376103

[23] Horner VL, Wolfner MF. Transitioning from egg to embryo: Triggers and mechanisms of egg activation. Dev Dyn. 2008;237: 527–544. 10.1002/dvdy.21454 18265018

[24] Wang QT, Piotrowska K, Ciemerych MA, Milenkovic L, Scott MP, Davis RW, et al A genome-wide study of gene activity reveals developmental signaling pathways in the preimplantation mouse embryo. Dev Cell. Cell Press; 2004;6: 133–144. 10.1016/S1534-5807(03)00404-0 14723853

[25] Aiken CEM, Swoboda PPL, Skepper JN, Johnson MH. The direct measurement of embryogenic volume and nucleo-cytoplasmic ratio during mouse pre-implantation development. Reproduction. 2004;128: 527–535. 10.1530/rep.1.00281 15509698

[26] Szollosi D, Calarco P, Donahue RP. Absence of centrioles in the first and second meiotic spindles of mouse oocytes. J Cell Sci. 1972;11: 521–541. 5076360 10.1242/jcs.11.2.521

[27] Clift D, Schuh M. A three-step MTOC fragmentation mechanism facilitates bipolar spindle assembly in mouse oocytes. Nat Commun. Nature Publishing Group; 2015;6: 1–12. 10.1038/ncomms8217 26147444 PMC4501430

[28] Madgwick S, Jones KT. How eggs arrest at metaphase II: MPF stabilisation plus APC/C inhibition equals Cytostatic Factor. Cell Div. BioMed Central; 2007;2: 4 10.1186/1747-1028-2-4 17257429 PMC1794241

[29] Wang QT, Piotrowska K, Ciemerych MA, Milenkovic L, Scott MP, Davis RW, et al A genome-wide study of gene activity reveals developmental signaling pathways in the preimplantation mouse embryo. Dev Cell. 2004;6: 133–144. 10.1016/S1534-5807(03)00404-0 14723853

[30] Gueth-Hallonet C, Antony C, Aghion J, Santa-Maria A, Lajoie-Mazenc I, Wright M, et al gamma-Tubulin is present in acentriolar MTOCs during early mouse development. J Cell Sci. 1993;105: 157–66. 8360270 10.1242/jcs.105.1.157

[31] Courtois A, Schuh M, Ellenberg J, Hiiragi T. The transition from meiotic to mitotic spindle assembly is gradual during early mammalian development. J Cell Biol. 2012;198: 357–370. 10.1083/jcb.201202135 22851319 PMC3413348

[32] Kubiak JZ, Chesnel F, Richard-Parpaillon L, Bazile F, Pascal A, Polanski Z, et al Temporal regulation of the first mitosis in Xenopus and mouse embryos. Mol Cell Endocrinol. 2008;282: 63–69. 10.1016/j.mce.2007.11.023 18178304

[33] Ajduk A, Strauss B, Pines J, Zernicka-Goetz M. Delayed APC/C activation extends the first mitosis of mouse embryos. Sci Rep. Nature Publishing Group; 2017;7: 9682 10.1038/s41598-017-09526-1 28851945 PMC5575289

[34] Crozet N, Dahirel M, Chesne P. Centrosome inheritance in sheep zygotes: Centrioles are contributed by the sperm. Microsc Res Tech. 2000;49: 445–450. 10.1002/(SICI)1097-0029(20000601)49:5<445::AID-JEMT6>3.0.CO;2-B 10842371

[35] Schatten H, Sun QY. The role of centrosomes in mammalian fertilization and its significance for ICSI. Mol Hum Reprod. 2009;15: 531–538. 10.1093/molehr/gap049 19549764 PMC2734160

[36] Manandhar G, Simerly C, Schatten G. Highly degenerated distal centrioles in rhesus and human spermatozoa. Hum Reprod. 2000;15: 256–263. 10.1093/humrep/15.2.256 10655294

[37] Sathananthan AH, Kola I, Osborne J, Trounson A, Ng SC, Bongso A, et al Centrioles in the beginning of human development. Proc Natl Acad Sci U S A. National Academy of Sciences; 1991;88: 4806–10. 10.1073/PNAS.88.11.4806 2052559 PMC51755

[38] Sathananthan AH, Ratnam SS, Ng SC, Tarín JJ, Gianaroli L, Trounson A. The sperm centriole: Its inheritance, replication and perpetuation in early human embryos. Hum Reprod. 1996;11: 345–356. 10.1093/HUMREP/11.2.345 8671223

[39] Fishman EL, Jo K, Nguyen QPH, Kong D, Royfman R, Cekic AR, et al A novel atypical sperm centriole is functional during human fertilization. Nat Commun. Springer US; 2018;9: 2210 10.1038/s41467-018-04678-8 29880810 PMC5992222

[40] Fitzharris G. A shift from kinesin 5-dependent metaphase spindle function during preimplantation development in mouse. Development. 2009;136: 2111–2119. 10.1242/dev.035089 19465601 PMC2730398

[41] Chaigne A, Campillo C, Voituriez R, Gov NS, Sykes C, Verlhac M-H, et al F-actin mechanics control spindle centring in the mouse zygote. Nat Commun. Nature Publishing Group; 2016;7: 10253 10.1038/ncomms10253 26727405 PMC4725770

[42] Reichmann J, Nijmeijer B, Hossain MJ, Eguren M, Schneider I, Politi AZ, et al Dual-spindle formation in zygotes keeps parental genomes apart in early mammalian embryos. Science. American Association for the Advancement of Science; 2018;361: 189–193. 10.1126/science.aar7462 30002254

[43] Harashima H, Dissmeyer N, Schnittger A. Cell cycle control across the eukaryotic kingdom. Trends Cell Biol. Elsevier Ltd; 2013;23: 345–356. 10.1016/j.tcb.2013.03.002 23566594

[44] Araujo AR, Gelens L, Sheriff RSM, Santos SDM. Positive Feedback Keeps Duration of Mitosis Temporally Insulated from Upstream Cell-Cycle Events. Mol Cell. Elsevier; 2016;64: 362–375. 10.1016/j.molcel.2016.09.018 27768873 PMC5077699

[45] Meraldi P, Draviam VM, Sorger PK. Timing and checkpoints in the regulation of mitotic progression. Dev Cell. Cell Press; 2004;7: 45–60. 10.1016/j.devcel.2004.06.006 15239953

[46] Musacchio A, Salmon ED. The spindle-assembly checkpoint in space and time. Nat Rev Mol Cell Biol. Nature Publishing Group; 2007;8: 379–393. 10.1038/nrm2163 17426725

[47] Rieder CL, Schultz A, Cole R, Sluder G. Anaphase onset in vertebrate somatic cells is controlled by a checkpoint that monitors sister kinetochore attachment to the spindle. J Cell Biol. Rockefeller University Press; 1994;127: 1301–10. 10.1083/JCB.127.5.1301 7962091 PMC2120267

[48] Musacchio A. The Molecular Biology of Spindle Assembly Checkpoint Signaling Dynamics. Curr Biol. Cell Press; 2015;25: R1002–R1018. 10.1016/J.CUB.2015.08.051 26485365

[49] Liu S-T, Zhang H. The mitotic checkpoint complex (MCC): looking back and forth after 15 years. AIMS Mol Sci. NIH Public Access; 2016;3: 597–634. 10.3934/molsci.2016.4.597 28920074 PMC5597056

[50] Hampl A, Eppig JJ. Translational regulation of the gradual increase in histone H1 kinase activity in maturing mouse oocytes. Mol Reprod Dev. Wiley-Blackwell; 1995;40: 9–15. 10.1002/mrd.1080400103 7702874

[51] Winston NJ. Stability of cyclin B protein during meiotic maturation and the first mitotic cell division in mouse oocytes. Biol Cell. Wiley/Blackwell (10.1111); 1997;89: 211–219. 10.1111/j.1768-322X.1997.tb01009.x 9429304

[52] Kotani T, Yasuda K, Ota R, Yamashita M. Cyclin B1 mRNA translation is temporally controlled through formation and disassembly of RNA granules. J Cell Biol. The Rockefeller University Press; 2013;202: 1041–55. 10.1083/jcb.201302139 24062337 PMC3787373

[53] Davydenko O, Schultz RM, Lampson MA. Increased CDK1 activity determines the timing of kinetochore-microtubule attachments in meiosis I. J Cell Biol. Rockefeller University Press; 2013;202: 221–9. 10.1083/jcb.201303019 23857768 PMC3718970

[54] Wassmann K, Niault T, Maro B. Metaphase I Arrest upon Activation of the Mad2-Dependent Spindle Checkpoint in Mouse Oocytes. Curr Biol. Cell Press; 2003;13: 1596–1608. 10.1016/J.CUB.2003.08.052 13678590

[55] Brunet S, Pahlavan G, Taylor S, Maro B. Functionality of the spindle checkpoint during the first meiotic division of mammalian oocytes. Reproduction. Society for Reproduction and Fertility; 2003;126: 443–50. 10.1530/REP.0.1260443 14525526

[56] Hoffmann S, Maro B, Kubiak JZ, Polanski Z. A Single Bivalent Efficiently Inhibits Cyclin B1 Degradation and Polar Body Extrusion in Mouse Oocytes Indicating Robust SAC during Female Meiosis I. Prigent C, editor. PLoS ONE. Public Library of Science; 2011;6: e27143 10.1371/journal.pone.0027143 22125605 PMC3220673

[57] McGuinness BE, Anger M, Kouznetsova A, Gil-Bernabé AM, Helmhart W, Kudo NR, et al Regulation of APC/C Activity in Oocytes by a Bub1-Dependent Spindle Assembly Checkpoint. Curr Biol. Cell Press; 2009;19: 369–380. 10.1016/j.cub.2009.01.064 19249208

[58] Homer HA, McDougall A, Levasseur M, Yallop K, Murdoch AP, Herbert M. Mad2 prevents aneuploidy and premature proteolysis of cyclin B and securin during meiosis I in mouse oocytes. Genes Dev. Cold Spring Harbor Laboratory Press; 2005;19: 202–7. 10.1101/gad.328105 15655110 PMC545877

[59] Gui L, Homer H. Spindle assembly checkpoint signalling is uncoupled from chromosomal position in mouse oocytes. Development. 2012;139: 1941–1946. 10.1242/dev.078352 22513372 PMC3347686

[60] Kolano A, Brunet S, Silk AD, Cleveland DW, Verlhac M-H. Error-prone mammalian female meiosis from silencing the spindle assembly checkpoint without normal interkinetochore tension. Proc Natl Acad Sci. National Academy of Sciences; 2012;109: E1858–E1867. 10.1073/pnas.1204686109 22552228 PMC3390881

[61] Lane SIR, Yun Y, Jones KT. Timing of anaphase-promoting complex activation in mouse oocytes is predicted by microtubule-kinetochore attachment but not by bivalent alignment or tension. Development. Oxford University Press for The Company of Biologists Limited; 2012;139: 1947–55. 10.1242/dev.077040 22513370

[62] Yoshida S, Kaido M, Kitajima TS. Inherent Instability of Correct Kinetochore-Microtubule Attachments during Meiosis I in Oocytes. Dev Cell. Cell Press; 2015;33: 589–602. 10.1016/j.devcel.2015.04.020 26028219

[63] Kyogoku H, Kitajima TS. Large Cytoplasm Is Linked to the Error-Prone Nature of Oocytes. Dev Cell. Elsevier Inc.; 2017;41: 287–298.e4. 10.1016/j.devcel.2017.04.009 28486131

[64] Tsurumi C, Hoffmann S, Geley S, Graeser R, Polanski Z. The spindle assembly checkpoint is not essential for CSF arrest of mouse oocytes. J Cell Biol. Rockefeller University Press; 2004;167: 1037–1050. 10.1083/jcb.200405165 15611331 PMC2172623

[65] Shoji S, Yoshida N, Amanai M, Ohgishi M, Fukui T, Fujimoto S, et al Mammalian Emi2 mediates cytostatic arrest and transduces the signal for meiotic exit via Cdc20. EMBO J. 2006;25: 834–845. 10.1038/sj.emboj.7600953 16456547 PMC1383546

[66] Madgwick S, Hansen D V, Levasseur M, Jackson PK, Jones KT. Mouse Emi2 is required to enter meiosis II by reestablishing cyclin B1 during interkinesis. J Cell Biol. Rockefeller University Press; 2006;174: 791–801. 10.1083/jcb.200604140 16966421 PMC2064334

[67] Kubiak JZ, Bazile F, Pascal A, Richard-Parpaillon L, Polanski Z, Ciemerych MA, et al Temporal regulation of embryonic M-phases. Folia Histochem Cytobiol. 2008;46: 5–9. 10.2478/v10042-008-0001-z 18296258

[68] Ciemerych MA, Maro B, Kubiak JZ. Control of duration of the first two mitoses in a mouse embryo. Zygote. 1999;7: 293–300. 10.1017/S0967199499000696 10717947

[69] Palmer N, Kaldis P. Regulation of the Embryonic Cell Cycle During Mammalian Preimplantation Development. Curr Top Dev Biol. Academic Press; 2016;120: 1–53. 10.1016/bs.ctdb.2016.05.001 27475848

[70] Wei Y, Multi S, Yang C-R, Ma J, Zhang Q-H, Wang Z-B, et al Spindle Assembly Checkpoint Regulates Mitotic Cell Cycle Progression during Preimplantation Embryo Development. Wang H, editor. PLoS One. Public Library of Science; 2011;6: e21557 10.1371/journal.pone.0021557 21720555 PMC3123354

[71] Jacobs K, Van de Velde H, De Paepe C, Sermon K, Spits C. Mitotic spindle disruption in human preimplantation embryos activates the spindle assembly checkpoint but not apoptosis until Day 5 of development. MHR Basic Sci Reprod Med. Oxford University Press; 2017;23: 321–329. 10.1093/molehr/gax007 28159965

[72] Dobles M, Liberal V, Scott ML, Benezra R, Sorger PK. Chromosome Missegregation and Apoptosis in Mice Lacking the Mitotic Checkpoint Protein Mad2. Cell. Cell Press; 2000;101: 635–645. 10.1016/S0092-8674(00)80875-2 10892650

[73] Kalitsis P, Earle E, Fowler KJ, Choo KH. Bub3 gene disruption in mice reveals essential mitotic spindle checkpoint function during early embryogenesis. Genes Dev. Cold Spring Harbor Laboratory Press; 2000;14: 2277–82. 10.1101/GAD.827500 10995385 PMC316933

[74] Wang Q, Liu T, Fang Y, Xie S, Huang X, Mahmood R, et al BUBR1 deficiency results in abnormal megakaryopoiesis. Blood. American Society of Hematology; 2004;103: 1278–85. 10.1182/blood-2003-06-2158 14576056

[75] Sikora-Polaczek M, Hupalowska A, Polanski Z, Kubiak JZ, Ciemerych MA. The First Mitosis of the Mouse Embryo Is Prolonged by Transitional Metaphase Arrest1. Biol Reprod. Oxford University Press; 2006;74: 734–743. 10.1095/biolreprod.105.047092 16382027

[76] Morris SA, Guo Y, Zernicka-Goetz M. Developmental Plasticity Is Bound by Pluripotency and the Fgf and Wnt Signaling Pathways. Cell Rep. Cell Press; 2012;2: 756–765. 10.1016/j.celrep.2012.08.029 23041313 PMC3607220

[77] Strnad P, Gunther S, Reichmann J, Krzic U, Balazs B, de Medeiros G, et al Inverted light-sheet microscope for imaging mouse pre-implantation development. Nat Methods. 2016;13: 139–42. 10.1038/nmeth.3690 26657559

[78] Gu B, Posfai E, Rossant J. Efficient generation of targeted large insertions by microinjection into two-cell-stage mouse embryos. Nat Biotechnol. Nature Publishing Group; 2018;36: 632–637. 10.1038/nbt.4166 29889212

